# Human Liver Macrophage Subsets Defined by CD32

**DOI:** 10.3389/fimmu.2020.02108

**Published:** 2020-09-23

**Authors:** Xia Wu, Nicole Hollingshead, Jessica Roberto, Allison Knupp, Heidi Kenerson, Antony Chen, Ian Strickland, Helen Horton, Raymond Yeung, Radika Soysa, Ian N. Crispe

**Affiliations:** ^1^Departments of Pathology, University of Washington Medical Center, Seattle, WA, United States; ^2^Departments of Surgery, University of Washington Medical Center, Seattle, WA, United States; ^3^Janssen Research and Development, Beerse, Belgium

**Keywords:** human, liver, myeloid, Kupffer cell, CD32

## Abstract

Human liver myeloid cells are imperfectly defined, but it is broadly agreed that cells of stellate appearance *in situ*, expressing the markers CD11b and CD68, are the liver's resident macrophages, classically termed Kupffer cells. Recent investigations using single cell RNA sequencing and unsupervised clustering algorithms suggest there are two populations of cells with the characteristics of tissue macrophages in human liver. We therefore analyzed dissociated human liver tissue using the markers CD11b and CD68 to define macrophage-like cells and found within this population two subsets that differ in their expression of multiple surface markers. These subsets were FACS-sorted based on CD32 expression, and gene expression analysis identified them with human liver myeloid cell subsets that were previously defined by two independent single cell RNA sequencing studies. Using qRT-PCR we found that the two subsets differed in the expression of genes associated with T cell activation and immunosuppression, suggesting distinct roles in T cell tolerance. In addition, one subset expressed two markers, CD1C and CD11c, more often seen on classical dendritic cells. Criteria used to distinguish macrophages from dendritic cells in other tissues may need to be revised in the human liver.

## Introduction

Multiple, independent lines of evidence suggest that there are two populations of macrophages in the mouse and in the human liver, as well as other tissues. In mice, these populations are distinguished by their transcriptome (1–3), by anatomical location (4) and by origin (5–8). There is a subset derived from the yolk sac and other early fetal progenitors (9), while other liver macrophages are the differentiation products of peripheral blood monocytes (2, 7). It is undecided whether the term Kupffer cells should apply only to the fetal-derived cells, or to both subsets. Here we will use the term to indicate all fully mature tissue macrophages, but this is not intended to pre-judge the issue. In mouse these are CD11b+ F4/80+ myeloid cells (10, 11), while in the human their markers include CD68 (12–14).

We chose to use CD68 and CD11b to define fully mature macrophages in the human liver. CD68 has good credentials in this regard (15). The terminology concerning these cells is in flux. Some maintain that Kupffer cells should be used only for cells presumed to be of embryonic origin and capable of local self-renewal, while any other cells should be termed “liver macrophages.” However, we don't think that embryo-derived macrophages can be unequivocally identified in human liver, either using surface staining or via gene expression (16). These cells are identified in the mouse only by using transgenic lineage-tracing approaches, and equating them to one of the two macrophage-like cell populations found in human liver by multiple methods (both using scRNAseq, and in our analysis by CD14 vs. CD32 staining) is an extrapolation and seems to us premature. Therefore, we follow the long-established usage that CD11b+ cells are myeloid, CD68+ cells are mature macrophages, and mature macrophages in the liver should all be called Kupffer cells. Others have defined all human liver CD68+ CD14+ cells as Kupffer cells and that is compatible with our working definition (17). Within Kupffer cells we recognize that there is heterogeneity and consider that this might result from embryonic vs. adult hematopoietic origin, but in human we cannot know this.

In the human, two recent single cell RNA sequencing (scRNAseq) studies reach the conclusion that there are two subsets of such liver macrophages (18, 19). A limitation of such studies is that while they allow cell function to be predicted from the expression of different genes and the identification of active signaling pathways, they do not directly allow function to be tested. This depends on the capacity to isolate the cells using markers expressed on the surface of intact, viable cells.

We have addressed the issue of human liver macrophage/Kupffer cell heterogeneity starting with the isolation of leukocytes from human liver tissue, obtained from tissue donors undergoing the resection of individual liver lobes. While we analyzed samples of tissue with no obvious disease, the tissue donors were not completely healthy. Most were carrying metastatic cancer from a colorectal primary tumor, in the form of a localized lesion confined to a single liver lobe. Patients with current or former infection with HBV, HCV, and HIV were excluded. Only a minority of the liver tissue samples were fibrotic.

Using a set of classic markers to define human liver macrophages and then exploring the expression of other myeloid cell surface markers we concluded that, while there is considerable individual variation, two subsets of Kupffer cells were reproducibly present, defined as CD14-high, CD32-mid and CD14-mid, CD32-high. These subsets were FACS-sorted based on CD32 expression alone, and their gene expression was evaluated to determine whether they correspond to the subsets recently identified using scRNAseq.

## Materials and Methods

### Human Liver Specimens

Fresh liver tissues were obtained from patients undergoing liver resection at the University of Washington Medical Center (Seattle, WA, USA). All patients in this study prospectively consented to donate liver tissue for research under the Institutional Review Board protocol #00001852. Clinical details of these patients are provided in Supplementary Table 1. A total of 26 liver specimens were analyzed in this study. Not all were analyzed in the same way, as explained in the Figure legends.

### Liver Slice Dissociation

Resected liver cores of 6 mm diameter were sliced to 250 μm thickness with the Leica VT1200 S vibratome (Nussloch, Germany) as described previously (20). Ten to fifteen liver slices were pooled and digested with the Type IV collagenase (Sigma-Aldrich, C5138) with the concentration of 2 mg/ml in Gey's Balanced Salt Solution (GBSS, Sigma-Aldrich, G9779), using one slice to 1.0 ml buffer in a 37°C water bath for 25 min. Slices were pipetted up-and-down at the 15 and 25 min time point to facilitate the dissociation of liver cells.

The digestion mixture was filtered through 100 μm pore-size cell strainers (VWR, 10199-659). Differential centrifugation was used to obtain liver cell populations enriched with non-parenchymal cells (21). Briefly, the cell suspension was centrifuged at 50 × *g* for 3 min at 4°C. The collected supernatants were further centrifuged at 500 × *g* for 5 min at 4°C. The cell pellets were resuspended with 10 ml GBSS, and were centrifuged again at 500 × *g* for 5 min at 4°C. The cell pellets were resuspended with 1.0 ml ice-cold PBS. Ten microlitre of the extracted cells were used for Trypan blue staining to evaluate cell viability, and total live cell numbers were determined using a hemocytometer.

### FACS Analysis of Myeloid Cells

The extracted liver cells were centrifuged again at 500 × *g* for 5 min at 4°C, and resuspended with 250 μl of PBS flow buffer (FBS buffer) [PBS, pH 7.4, 1 mM EDTA, 2% FBS] (21). Five microlitre of Fc receptor blocking solution (Biolegend, cat # 422302, 1:50 v/v ratio) was added to the cell mixture to each sample and incubate on ice for 30 min. After the incubation, 20 μl of cell mixture solution was collected as the unstained controls. The remaining samples were analyzed with antibody cocktails for surface staining, as described in Tables 1–3. Typically, 1 μl of each antibody was added to each sample, i.e., the antibody: sample is 1:250 (v/v). The Live/Dead fixable blue dead cell stain (Invitrogen, cat # L23105) was used at a final concentration of 1: 500. Staining mixture was incubated on ice for 30 min. Tubes were tapped every 10 min to help antibody binding. After the incubation, cells were pelleted with 500 × *g* for 5 min at 4°C, and cells were washed twice with 1.0 ml of FBS buffer each time.

**Table 1:** Staining panel including CD16 among myeloid markers, grouping all DC markers in APC.

	**Antibody target**	**Available source**	**Catalog #**	**Clone**	**Fluorophore**
1	Anti-CD3	Biolegend	317329	OKT3	BV785
2	Anti-CD45	Biolegend	304023	HI30	Alexa Fluor 700
3	Anti-CD11b	Biolegend	101217	M1/70	Alexa Fluor 488
4	Anti-CD68	Biolegend	333821	Y1/82A	APC-Cy7
5	Anti-CD32	Biolegend	303205	FUN-2	PE
6	Anti-CD14	Biolegend	367111	63D3	PE-Cy7
7	Anti-CD206	Biolegend	321121	15-2	PerCP-Cy5.5
8	Anti-CD16	eBioscience	48-0168-42	eBioCB16 (CB16)	eFluor 450 (BV421)
9	Anti-CD1c + Anti-CD303 + Anti-CD141	all Miltenyi	130-110-595 + 130-113-752 + 130-113-876	REA694 + AC144 + AD5-14H12	all APC
10	Live/Dead staining	Thermo-Fisher	L23105	n/a	UV-DAPI

**Table 2:** Staining panel including CD206 and the three classic human DC markers, BDCA-1 (CD1c), BDCA-1 (CD303), and BDCA-3 (CD141).

	**Antibody target**	**Source**	**Catalog #**	**Clone**	**Fluorophore**
1	Anti-CD3	Biolegend	317329	OKT3	BV785
2	Anti-CD45	Biolegend	304023	HI30	Alexa Fluor 700
3	Anti-CD11b	Biolegend	101217	M1/70	Alexa Fluor 488
4	Anti-CD68	Biolegend	333821	Y1/82A	APC-Cy7
5	Anti-CD14	Biolegend	367111	63D3	PE-Cy7
6	Anti-CD206	Biolegend	321121	15-2	PerCP-Cy5.5
7	Anti-CD1c (BDCA-1)	Miltenyi	130-110-594	REA694	PE
8	Anti-CD303 (BDCA-2)	Miltenyi	130-106-505	AC144	VioBlue (BV421)
9	Anti-CD141 (BDCA-3)	Miltenyi	130-113-876	AD5-14H12	APC
10	Live/Dead staining	Thermo-Fisher	L23105	n/a	UV-DAPI

**Table 3:** Antibody staining panel to identify CD1C, CD141, and CD11c staining on human myeloid subsets.

	**Antibody target**	**Source**	**Catalog #**	**Clone**	**Fluorophore**
1	Anti-CD3	Biolegend	317329	OKT3	BV785
2	Anti-CD45	Biolegend	304023	HI30	Alexa Fluor 700
3	Anti-CD11b	Biolegend	101217	M1/70	Alexa Fluor 488
4	Anti-CD68	Biolegend	333821	Y1/82A	APC-Cy7
5	Anti-CD14	Biolegend	367111	63D3	PE-Cy7
6	Anti-CD32	Biolegend	303215	FUN-2	PerCP-Cy5.5
7	Anti-CD1c (BDCA-1)	Miltenyi	130-110-594	REA694	PE
8	Anti-CD141 (BDCA-3)	Miltenyi	130-113-876	AD5-14H12	APC
9	Anti-CD11c	BD Biosciences	562561	B-ly6	BV421
10	Live/Dead staining	Thermo-Fisher	L23105	n/a	UV-DAPI

For the subsequent intracellular staining analysis (6), cell pellets were resuspended with 250 μl of BD cytofix/Cytoperm (BD Bioscience, cat # 554722) at 4°C. Cells were pelleted with 500 × *g* for 7 min at 4°C and were washed twice with 1.0 ml of BD Perm/Wash buffer (BD Bioscience, cat # 554723). Cell pellets were resuspended using 50 μl of Perm/Wash buffer, 1 μl of anti-CD68 antibody (Biolegend, cat # 333822) was added to each sample, and samples were incubated with another 30 min on ice. Tubes were tapped every 10 min to help antibody binding. Cells were pelleted with 500 × *g* for 8 min at 4°C and were washed twice with 1.0 ml of BD Perm/Wash buffer. Cell pellets were resuspended with 400 μl FBS buffer for FACS analysis using an LSRII cytometer (BD Biosciences) at the Pathology Flow Cytometry Core Facility at University of Washington. The FACS data were analyzed using FlowJo software (Treestar, version 10.5.0). Cells were gated for single cells and live cells, then to identify the CD45+CD3- population.

### Gene Expression Analysis for CD32-Mid and CD32-High Cells

**Figure 1:**
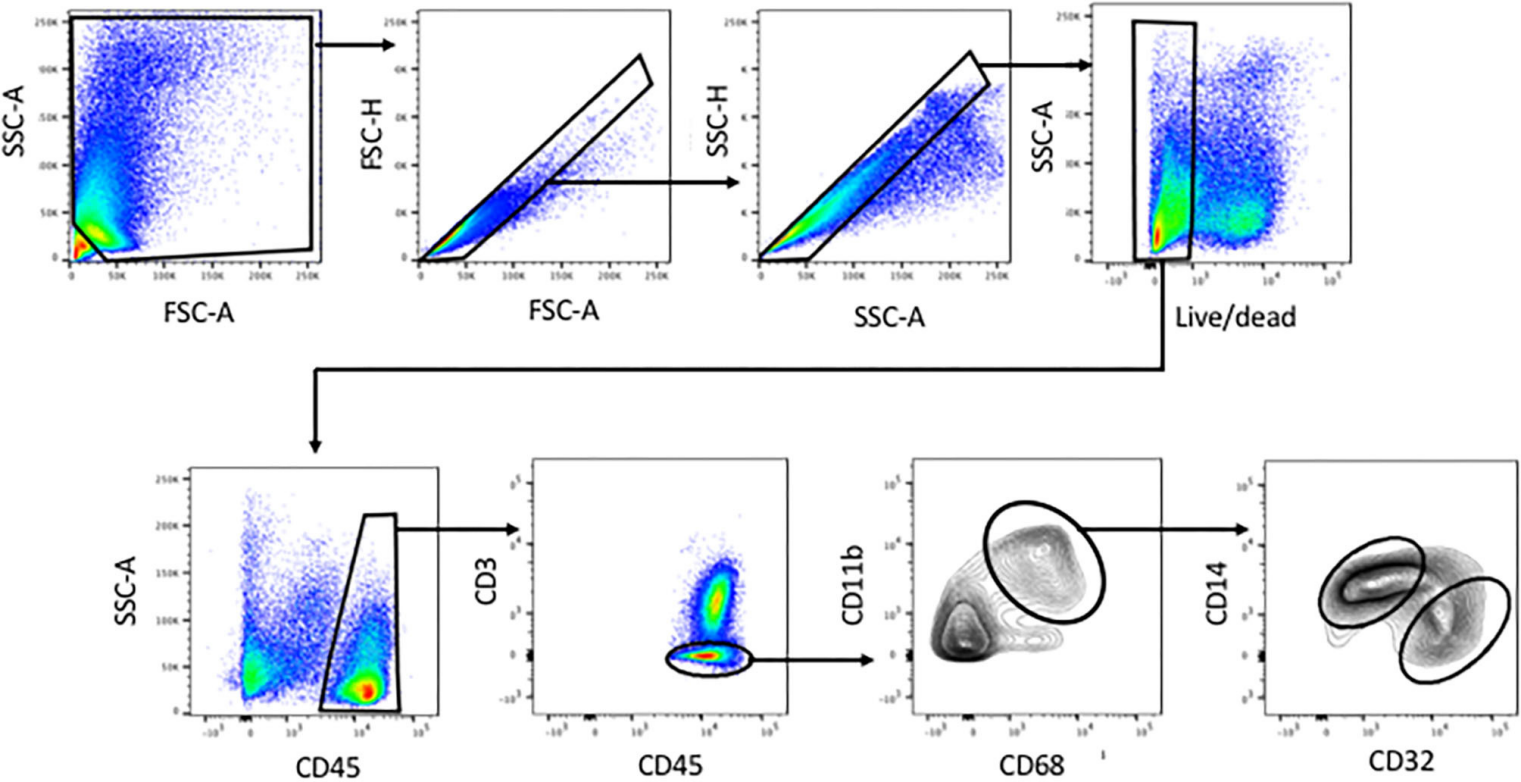
Identification of human liver myeloid cells, Kupffer cells and Kupffer cell subsets within the viable single cells harvested from collagenase-digested human liver tissue. Cells were first gated using FSC-A and SSC-A to exclude sub-cellular debris, then sequentially using FSC-H vs. FSC-A and SSC-H vs. SSC-A to exclude doublets. A live-dead stain was used to exclude cells with damaged membranes. Subsequent analysis involved gating on CD45+ cells, then on CD3-negative cells, then on CD11b+ CD68+ cells. Within the CD11b+ CD68+ subset, we invariably found CD14-high CD32-mid cells and CD14-mid, CD32-high cells. Analyses based on this gating scheme were performed on 23 separate patient tissue samples.

**Figure 2:**
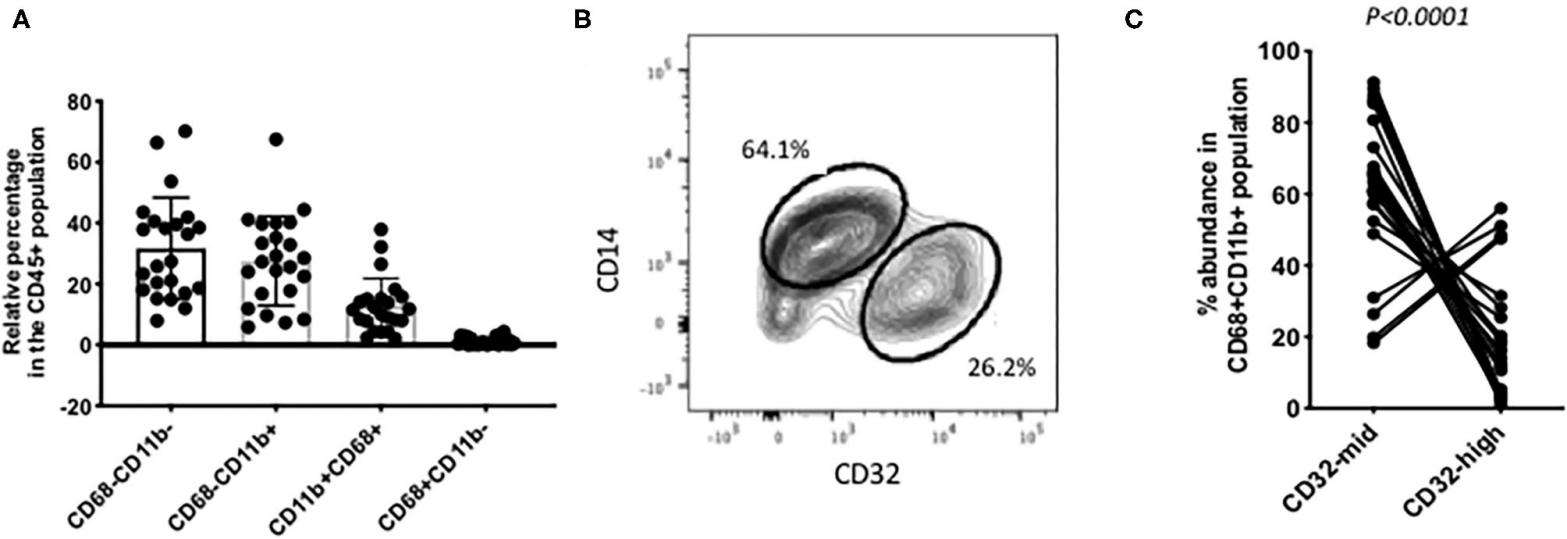
Individual variation in myeloid cell subsets. **(A)** Among the CD45+ CD3– liver cells, the most abundant populations were lacking CD68. Around half of those were other myeloid cells, based on their expression of CD11b. Putative Kupffer cells, CD11b+ CD68+ were variable in abundance from 2% up to 40% of the total. Cells with CD68 but no CD11b were present in most samples but always 5% or less. **(B)** A frequent pattern of staining within the CD45+ CD11b+ CD68+ cells was that CD14-high, CD32-mid cells were in excess over CD14-mid, CD32-high cells. **(C)** Variation among 22 individual samples. The CD14-high, CD32-mid cells were most often between 60 and 100% of the total, and the CD12-mid, CD32-high cells correspondingly between 40% and none. The reproducibility of this imbalance was highly significant (*p* < 0.0001, paired Wilcoxon test). The data in A and C represent 22 separate patient samples.

**Figure 3:**
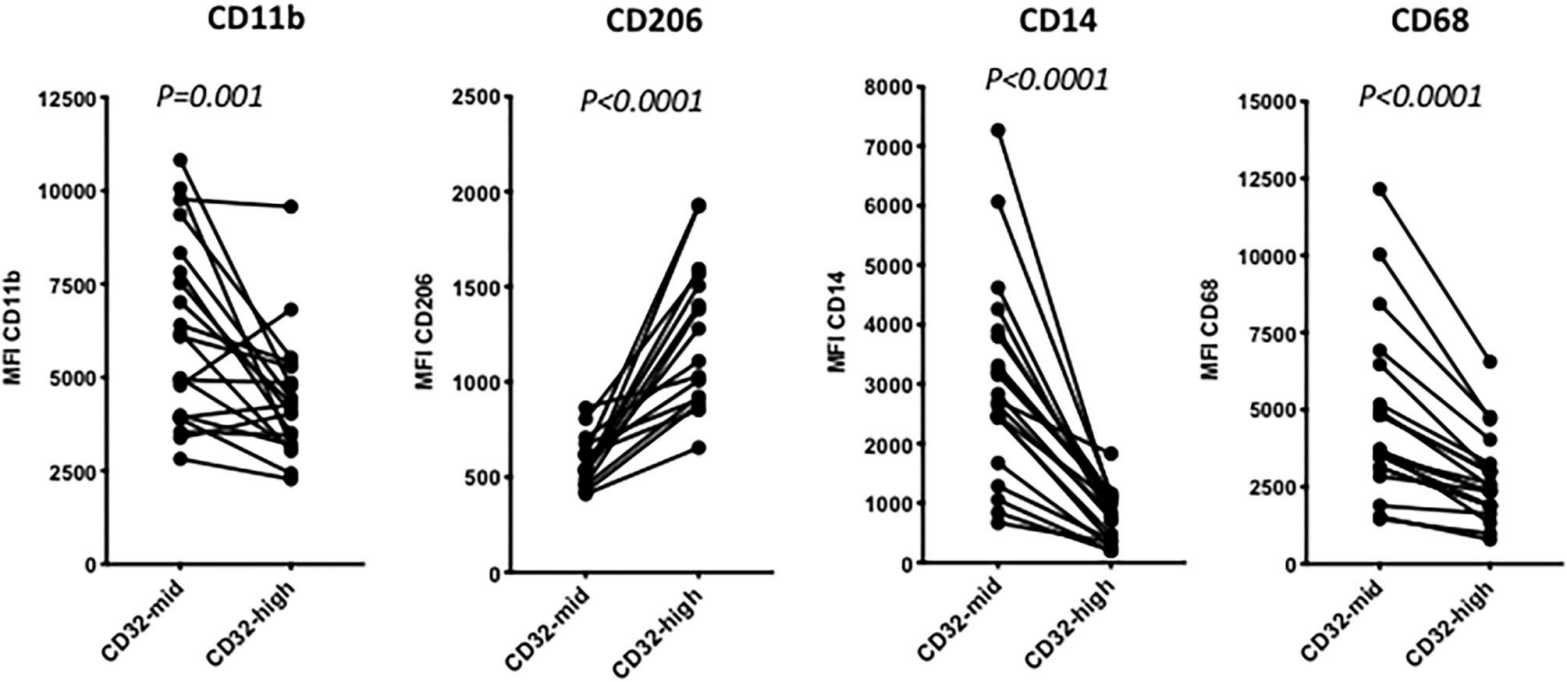
The distinction between 14-high, CD32-mid cells and CD14-mid, CD32-high cells was robust based on other markers. Thus, the CD14-mid, CD32-high cells expressed significantly lower CD11b, significantly higher CD206, significantly lower CD14 and also significantly lower CD68, based on MFI. The significance of differences was evaluated using a paired Wilcoxon test. The data represent 22 separate patient samples.

**Figure 4:**
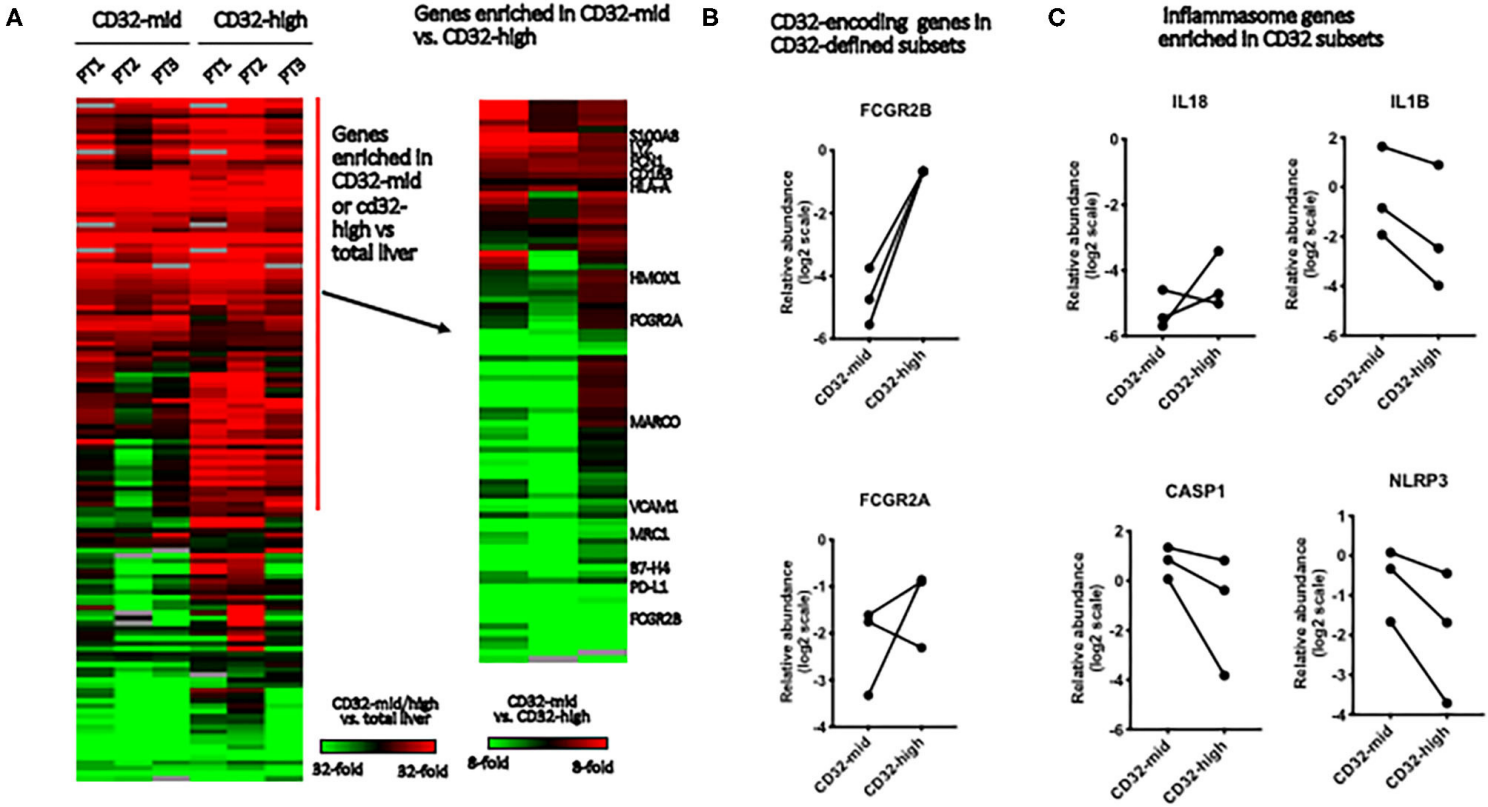
Gene expression in Kupffer cell subsets. We evaluated the differences between FACS-sorted CD45+ CD3– CD68+ CD32-high and CD45+ CD3– CD68+ CD32-mid cells, using microfluidic qRT-PCR to measure 96 target genes. These cells were isolated from tissue samples sufficiently large, and with permissive anatomy to allow perfusion of the tissue with collagenase. This allowed us to evaluate tissue from three separate patients. **(A)** Genes were first stratified to identify those expressed more highly in either FACS-sorted sample than in total liver, then this subset stratified to determine which genes were different between the subsets. **(B)** We tested expression of two genes that encode elements of the CD32 antigen. Expression of FCGR2B was perfectly correlated with cell surface expression of CD32, but FCGR2A was not reliably predicted by surface staining. **(C)** Of four inflammasome component-encoding genes tested, three were consistently expressed more strongly in the CD32-mid subset. Since we could obtain only 3 sets of paired samples, these data were not subjected to a significance test.

**Figure 5:**
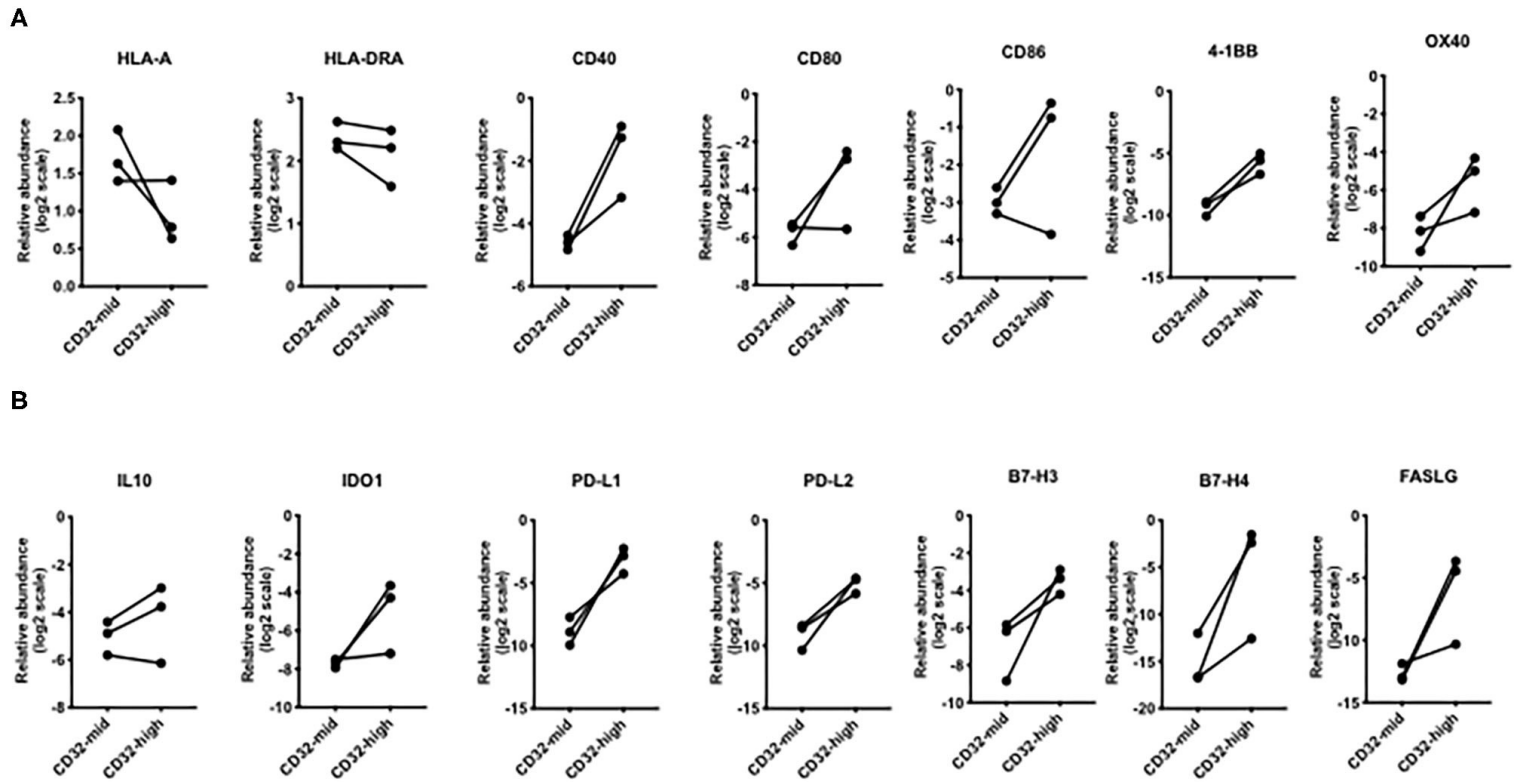
Differential expression of genes involved in T cell interactions. **(A)** Broadly, the CD32-high subset expressed lower levels of MHC genes, but higher levels of genes encoding multiple co-stimulatory molecules: *CD40, 4-1BB*, and *OX40*. **(B)** For every immunosuppressive gene except *IL-10*, the CD32-high cells consistently expressed a higher level than the CD32-mid cells. These included *PD-L1, PD-L2, B7-H3, B7-H4*, and *FASLG*. Since we could obtain only 3 sets of paired samples, these data were not subjected to a significance test.

**Figure 6:**
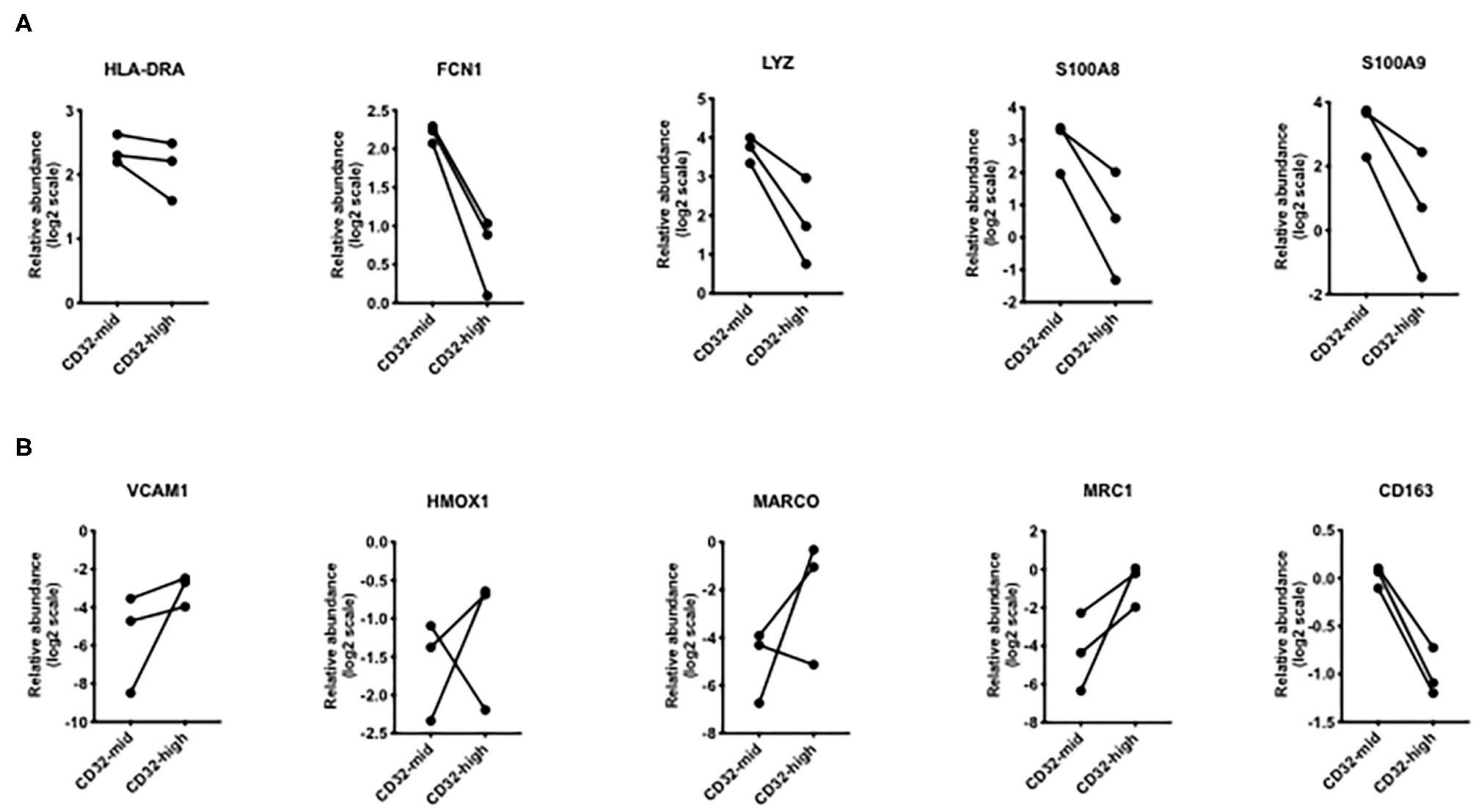
Differential expression of genes that were different in scRNAseq analyses. **(A)** Genes that were increased in “inflammatory” Kupffer cells were mostly higher in CD32-mid Kupffer cells. **(B)** Genes that were increased in “immunosuppressive” Kupffer cells were mostly higher in CD32-high Kupffer cells. Since we could obtain only 3 sets of paired samples, these data were not subjected to a significance test.

Three liver specimens were large enough for analysis with liver perfusion as described previously (20). Liver cells were stained and sorted using a BD FACS Aria III (BD Biosciences), and sub-population of myeloid cells with the phenotypes CD45+ CD3– CD68+ CD11b+ CD14+ CD32-mid or CD45+ CD3– CD68+ CD11b+ CD14+ CD32-high were collected. We did not discriminate levels of CD14 in this sort. The use of CD68 as a cell surface marker is complicated, since most of the molecule is intracellular and best revealed by staining permeabilized cells, as in Figures 1–3 and Supplementary Figures 1–4. However, we found enough CD68 staining on intact human liver cells to use this marker in Kupffer cell isolation, generating the data in Figures 4–6.

RNA was extracted from the cell pellets with populations with TRIzol and the Direct-zol RNA MiniPrep Kit (Zymo Research, Irvine, CA, USA) (20). The cDNA was synthesized with the QuantiTect Reverse Transcription Kit (Qiagen, Hilden, Germany). Gene expression was analyzed with a 48 × 48 dynamic array and a BioMark HD microfluidics system (Fluidigm, San Francisco, CA, USA). The Fluidigm Real-Time PCR Analysis software was used to calculate Ct thresholds, using the settings of quality threshold 0.65, baseline correction linear, Ct threshold method auto detection. Gene abundance in individual liver slices was normalized to the averaged Ct values of ACTB, HPRT, and GAPDH.

### Statistical Significance Tests

The non-parametric Mann Whitney test and Wilcoxon matched-pairs signed rank test and Spearman correlation test were performed using Prism (version 8.1.2) (GraphPad Software Inc., CA, USA).

## Results

### Macrophages vs. Monocytes

Tissue dissociation and flow cytometry were used to define human liver myeloid cell subsets. We identified putative macrophages by size gating to eliminate doublets, the exclusion of dead cells using a cell permeability dye, and the presence of CD45 but the lack of CD3 (Figure 1). These cells were further stained for CD11b and CD68, and cells expressing both were defined as Kupffer cells. We always (*n* = 23) identified such cells, while other populations such as CD68+ CD11b- cells were present in some liver tissue samples (Figure 1). The putative Kupffer cells, expressing both CD11b and CD68, were relatively abundant but their frequency was highly variable, from 10 to 90% of all CD45+ CD3- cells, with a mean of 40%, while cells with CD68 but no CD11b were rare, around 0–5% (Figure 2A). Within the population of CD68+ CD11b+ liver macrophages, two subsets were identified using the markers CD14 and CD32 (Figures 1, 2B). The cells with higher CD14 and lower CD32 were almost always more abundant, but the CD14-mid, CD32-high cells were always present (Figure 2C). These two subsets differed in other markers, and the differences in the expression of CD11b, CD206, CD14, and CD68 were highly consistent between the CD32-mid and the CD32-high cells (Figure 3).

We applied the same analytical tools to peripheral blood leukocytes, with very different results. Equivalent gating identified single, viable human peripheral blood CD45+ CD3- cells, which expressed a high level of CD11b and a low level of CD68. These cells however revealed only a single population, based on CD14 vs. CD32 staining (Supplementary Figure 1). Compared to liver-derived myeloid cells, these cells expressed a lower level of CD68, no CD206, and were unimodal and low for CD32 (Supplementary Figure 2A). These differences between blood and liver cells were consistent across 11–12 individual cell donors (Supplementary Figure 2B).

### Gene Expression in Liver Macrophage Subsets

To test the robustness of CD32 as a macrophage/Kupffer cell subset marker, we isolated the CD32-mid and CD32-high subsets of human liver CD45+ CD3– CD14+ CD68+ Kupffer cells from three human livers by FACS-sorting. A panel of 96 myeloid, macrophage or other cell type-specific genes was measured using qRT-PCR, and we focused on those expressed more strongly in both macrophage subsets than in other liver cells (Figure 4A). While the expression of *IL-18* was not consistent, three other inflammasome-linked genes, *IL1B, CASP1* and *NLRP3*, were more strongly expressed in the CD32-mid subset (Figure 4B).

We examined the expression of genes linked to immune function. While *HLA-A* was more strongly expressed by CD32-mid cells and *HLA-DRA* was indecisive, a number of co-stimulatory genes (*CD40, CD80, CD86, 4-1BB, OX40*) were generally expressed more strongly by the CD32-high cells (Figure 5A). Conversely, multiple co-inhibitory molecules were also expressed more strongly by the CD32-high cells (*PD-L1, PD-L2, B7-H3, B7-H4, FASLG*) (Figure 5B).

To test the hypothesis that the two liver macrophage subsets defined by CD32 were equivalent to those identified in scRNAseq experiments, we measured a set of the most discriminating genes from the studies of MacParland et al. (Figure 6). Among genes strongly expressed in cells termed “inflammatory Kupffer cells”, *FCN1, LYZ, S100A8*, and *S100A9* were more strongly expressed in the CD32-mid subset. Conversely, among genes more strongly expressed in cells identified as “tolerogenic Kupffer cells,” *VCAM1 HMOX1 MARCO* and *MRC1* were more strongly expressed in CD32-high cells. The gene *CD163* was out of step, however. It was documented by scRNAseq in the cells with these other markers, but our qRT-PCR from FACS-sorted cells showed that expression was consistently higher in CD32-mid cells.

**Figure 7:**
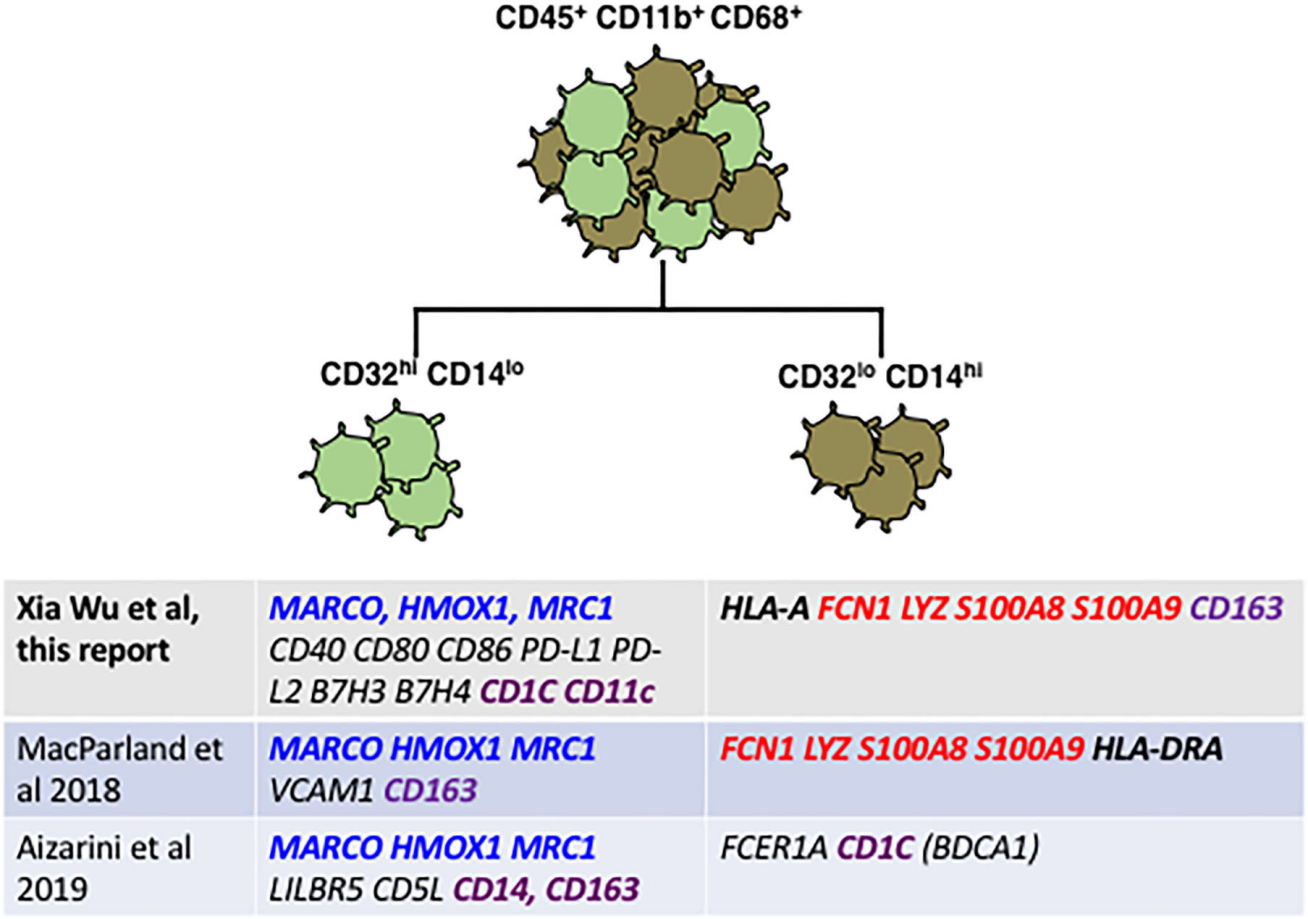
Reconciliation of our staining plus qRT-PCR data from FACS-sorted CD32-mid and CD32-high Kupffer cells with data from two published scRNAseq studies reveals broad agreement. Genes highlighted in blue seem to be diagnostic of a subset optimized for endocytosis and immune suppression, which genes highlighted in red are diagnostic for a subset optimized for inflammation and anti-microbial activity. Genes that are disparate between the different studies are highlighted in purple. Note in particular that CD163 expression in our study was discordant with the two single cell RNA sequencing studies.

Looking at the genes most divergent in two human liver cell scRNAseq papers, there is broad agreement between these marker-agnostic approaches and the data presented here using CD32 to identify subsets (Figure 7). All research groups find two subsets of human Kupffer cells, and the most conserved gene expression markers seem to be *FCN1, LYZ, S100A8* and *S100A9* in the CD32-mid cells, vs. *MARCO, HMOX1* and *MRC1* in the CD32-high cells.

We attempted to confirm the results of the gene expression analysis by surface staining for the protein encoded by the gene *MARCO*, which was one of the most divergent genes that was also differentially expressed in our study and the two scRNAseq papers. However, after testing several antibodies and diverse staining conditions we could not obtain satisfactory Marco cell surface staining.

In addition, we also measured the expression of a larger set of monocyte, macrophage, and lineage-related genes (Supplementary Table S2), but they were uninformative concerning Kupffer cell subset identity. We have uploaded the full dataset to the GEO database (accession number GSE154318).

## Discussion

Since there is not yet an agreed definition that distinguishes Kupffer cells from other macrophages in the liver, we use the term broadly for any fully mature macrophage, but we recognize that an agreed and more restrictive use of the term may emerge over time. Against that background, the data presented here argue that CD32 is a decisive cell surface marker for the identification of human liver macrophage/Kupffer cell subsets. The CD32 epitope is shared by the human Fc-gamma receptors type 2, encoded by three genes (22). The *FCGR2A* and *FCGR2C* genes encode activating receptors, while *FCGR2B* encodes an inhibitory receptor (23). Expression of CD32 increases as monocytes differentiate into macrophages under the influence of GM-CSF (24). Expression of CD32 on human monocytes may also be enhanced by IFN-gamma treatment, promoting antibody-dependent cellular cytotoxicity (25). CD32 is expressed and functional on several types of myeloid cells, including eosinophils (26), neutrophils (27), and a subset of human peripheral blood dendritic cells (28).

We do not think that a significant number of monocytes are present in our human liver myeloid cell samples. We addressed the issue of monocytes using CD11b vs. CD68 staining on peripheral blood mononuclear cells. This is shown in Supplementary Figure 1. We find that, indeed. blood monocytes expressed detectable CD68 but that all of the liver myeloid cells expressed a higher level (Supplementary Figure 2), consistent with differentiation from monocytes to mature macrophages. Also, liver CD68+ CD11b+ cells expressed CD206 and even the CD32-mid subset expressed more CD32 that did blood monocytes (Supplementary Figure 2). For this reason, we did not consider that we might be seeing abundant monocytes in human liver tissue. Many of the liver macrophage subset genes we evaluated are also expressed by monocytes, including *s100A8, s100A9, Lyz*, and *Fcn1*. However, beyond maturity as manifested by the level of CD68 expression, and markers such as CD206, there is no established Gold Standard to distinguish between monocytes and macrophages in human liver, so the strongest evidence comes from the expression of CD68, CD206, and CD32 by cells from the liver and not from the blood.

We included CD32 among the cell surface markers used to study human liver myeloid cell heterogeneity, and found that despite significant individual variation, it reliably distinguished two CD68+ myeloid cell subsets. Several surprising issues emerge from our analysis of gene expression in these two subsets. First, we found increased expression of both co-stimulatory and co-inhibitory genes in the CD32-high cells. This was not specifically identified in the scRNAseq studies (18, 19). It is possible that the expression of these functionally antagonistic genes is an artifact of the process of surface-staining and FACS-sorting the cells, but we favor the hypothesis that the CD32-high cells in the human tissue to which we have access are more strongly affected by the presence of cancer elsewhere in the same liver lobe, and this accounts for the strong expression of these immunologically active genes.

Second, the scRNAseq paper of Aizarini et al. (19) identified CD1C expression on the cells that most closely correspond to CD32-mid cells. CD1C is also known as blood dendritic cell antigen-1 (BDCA1), a classical marker for type 2 classical dendritic cells (cDC2). To gain insight into this potential overlap of Kupffer cells and cDC2, we stained CD68+ CD11b+ human liver cells to reveal a distinct subset of BDCA1+ cells. The frequency of these cells was correlated closely with the frequency of CD32-high cells (Supplementary Figure 3). We cannot say whether these Kupffer cells are equivalent to cDC2 in other tissue, or whether the expression of BDCA1 is a feature of macrophages in the liver environment. In addition to BDCA1, the CD32-high cells expressed significantly higher CD11c than did the CD32-mid cells (Supplementary Figure 4). However, the case that these cells are cDC2 is inconclusive; peripheral blood CD32+ dendritic cells were CD14-negative (28), arguing that they are not the same as the CD14-mid, CD32-high liver cells described here.

The difficulty of resolving macrophages from dendritic cells in the liver is also an issue in the mouse. Thus, one irradiation-reconstitution study defined a subset of sub-capsular radio-sensitive macrophages that were replaced by adult bone marrow precursors, expressed CX3CR1, and were dendritic in morphology (29). A separate study identified very similar cells as dendritic cells (2).

Although the CD68+, CD11b– subset of human liver myeloid cells were present in only a subset of the samples, we analyzed the expression of a subset of monocyte and macrophage markers on these cells (Supplementary Figure 5). When present, they were low to negative in the expression of CD14, CD16, CD32, and CD206, so we conclude that these cells were neither monocytes nor macrophages. Of most interest here. they expressed no markers characteristic of either Kupffer cell subset.

A recent study identified distinct populations of myeloid cells in the human fibrotic liver (30). Single cell RNAseq identified two clusters of cells the authors termed KC1 and KC2, which we think are the two normal Kupffer cells subsets we describe here. Both subsets became less abundant in fibrotic liver. These cell populations were quite distinct from each other based on unsupervised clustering analysis, and differed for example in *C1QC* gene expression, but there is insufficient data to assign them to either of the macrophage subsets represented in Figure 7. Both were different from tissue monocytes, identified in this study by *S100A12* and/or *MNDA* expression. There were also two myeloid cell subsets that were abundant among fibrotic liver non-parenchymal cells, and rare in the non-fibrotic livers. We would not expect to find such fibrosis-associated macrophages in the majority of our clinical samples, since patients with cirrhosis were most common among HCV+ and HBV+ liver tissue, both of which were excluded from the study. Most of the patients in our study did not have any liver fibrosis, but the CD14-mid, CD32-high and CD14-high, CD32-mid Kupffer cell subsets were present in almost all patients' liver tissue (Figure 2C). Since our study was not aimed to detect these fibrosis-associated cells but to define the macrophage populations in more normal liver tissue, the strongest evidence that we are not observing fibrosis-associated myeloid cells comes from the selection of a largely non-fibrotic group of tissue donors.

We do not know whether these two populations of liver macrophages are differentially located within the sinusoid, however these is reason to believe that niches occupied by liver macrophages are not homogeneous across the sinusoid. Concentration gradients of diverse metabolites may account for the differential location of subsets of hepatic stellate cells (31), while hepatic stellate cells in turn form part of the composite niche occupied by monocyte-derived macrophages, which imprints on them Kupffer cell identity (32).

In summary, we have identified two subsets of normal human liver macrophages using CD32 as the distinguishing cell surface marker. The gene expression in these two subsets is broadly consistent with subsets recently defined by scRNAseq, with the advantage that cells identified using cell surface markers may be FACS-sorted for future functional studies. The CD32-mid cells, which are also CD14-high, conform to all definitions of Kupffer cells. The CD32-high, CD14-mid cells are more ambiguous, since they express some markers more characteristic of cDC2 in other tissues. Functional experiments will more decisively resolve the biology of these provocative CD32-high macrophage-like cells.

Our study revealed significant individual variation in human liver myeloid cell populations. It is important to note that sources of “normal” human liver tissue are very limited. In our study, tissue was obtained from surgical resections that were performed for cancer, most often for metastatic colorectal cancer (Supplementary Table S1). While we studied myeloid cells in non-involved liver tissue, the tissue donors were not healthy. Outside of the tissue donors we studied here, a small number of potential donors also had co-incident chronic viral hepatitis, but anecdotally we did not observe any distinct changes in myeloid cells linked to these disease states. It would be valuable to extend the analysis first by collecting tissue from donors with distinct disease states in numbers sufficient to conduct an adequately powered study to see disease-linked phenotypes through the background noise, and to FACS-sort the cell subsets we identify to test their gene expression and biological function.

## Data Availability Statement

The datasets presented in this study can be found in online repositories. The names of the repository/repositories and accession number(s) can be found below: https://www.ncbi.nlm.nih.gov/geo/, GSE154318.

## Ethics Statement

The studies involving human participants were reviewed and approved by Institutional Review Board, UW Medicine. The patients/participants provided their written informed consent to participate in this study.

## Author Contributions

XW contributed to experimental design, performed the experiments, and analyzed data and wrote the manuscript. NH, JR, AK, and HK performed experiments. AC performed experiments and co-designed the study. IS and HH co-designed the study. RY led the clinical tissue harvest and co-wrote the manuscript. RS co-wrote the manuscript. IC designed the study, analyzed and interpreted data, and co-wrote the manuscript. All authors contributed to the article and approved the submitted version.

## Conflict of Interest

The work was supported, and three of the authors are employed, by Janssen Research and Development, a division of Johnson and Johnson, a pharmaceutical company. The remaining authors declare that the research was conducted in the absence of any commercial or financial relationships that could be construed as a potential conflict of interest.

## References

[1] Chakarov S Lim HY Tan L Lim SY See P Lum J. Two distinct interstitial macrophage populations coexist across tissues in specific subtissular niches. Science. (2019) 363:6432. 10.1126/science.aau0964 30872492

[2] David BA Rezende RM Antunes MM Santos MM Freitas Lopes MA Diniz AB. Combination of mass cytometry and imaging analysis reveals origin, location, and functional repopulation of liver myeloid cells in mice. Gastroenterology. (2016) 151:1176–91. 10.1053/j.gastro.2016.08.024 27569723

[3] Han X Wang R Zhou Y Fei L Sun H Lai S. Mapping the mouse cell atlas by microwell-seq. Cell. (2018) 172:1091–107.e17. 10.1016/j.cell.2018.02.001 29474909

[4] Sleyster EC Knook DL. Relation between localization and function of rat liver Kupffer cells. Lab Invest. (1982) 47:484–90. 6182391

[5] Hagemeyer N Kierdorf K Frenzel K Xue J Ringelhan M Abdullah Z. Transcriptome-based profiling of yolk sac-derived macrophages reveals a role for Irf8 in macrophage maturation. EMBO J. (2016) 35:1730–44. 10.15252/embj.201693801 27412700 PMC5010043

[6] Soysa R Lampert S Yuen S Douglass AN Li W Pfeffer K. Fetal origin confers radioresistance on liver macrophages via p21(cip1/WAF1). J Hepatol. (2019) 71:553–62. 10.1016/j.jhep.2019.04.015 31077791 PMC12509797

[7] Guillot A Tacke F. Liver macrophages: old dogmas and new insights. Hepatol Commun. (2019) 3:730–43. 10.1002/hep4.1356 31168508 PMC6545867

[8] Ginhoux F Guilliams M. Tissue-resident macrophage ontogeny and homeostasis. Immunity. (2016) 44:439–49. 10.1016/j.immuni.2016.02.024 26982352

[9] Sheng J Ruedl C Karjalainen K. Most tissue-resident macrophages except microglia are derived from fetal hematopoietic stem cells. Immunity. (2015) 43:382–93. 10.1016/j.immuni.2015.07.016 26287683

[10] Kinoshita M Uchida T Sato A Nakashima M Nakashima H Shono S. Characterization of two F4/80-positive Kupffer cell subsets by their function and phenotype in mice. J Hepatol. (2010) 53:903–10. 10.1016/j.jhep.2010.04.037 20739085

[11] Movita D Kreefft K Biesta P van Oudenaren A Leenen PJ Janssen HL. Kupffer cells express a unique combination of phenotypic and functional characteristics compared with splenic and peritoneal macrophages. J Leukoc Biol. (2012) 92:723–33. 10.1189/jlb.1111566 22685319

[12] Said EA Al-Reesi I Al-Riyami M Al-Naamani K Al-Sinawi S Al-Balushi MS. Increased CD86 but not CD80 and PD-L1 expression on liver CD68+ cells during chronic HBV infection. PLoS ONE. (2016) 11:e0158265. 10.1371/journal.pone.0158265 27348308 PMC4922653

[13] Kwekkeboom J Kuijpers MA Bruyneel B Mancham S De Baar-Heesakkers E Ijzermans JN. Expression of CD80 on Kupffer cells is enhanced in cadaveric liver transplants. Clin Exp Immunol. (2003) 132:345–51. 10.1046/j.1365-2249.2003.02129.x 12699427 PMC1808714

[14] Lapis K Zalatnai A Timar F Thorgeirsson UP. Quantitative evaluation of lysozyme- and CD68-positive Kupffer cells in diethylnitrosamine-induced hepatocellular carcinomas in monkeys. Carcinogenesis. (1995) 16:3083–5. 10.1093/carcin/16.12.3083 8603489

[15] Shan Z Ju C. Hepatic macrophages in liver injury. Front Immunol. (2020) 11:322. 10.3389/fimmu.2020.00322 32362892 PMC7180226

[16] Bleriot C Ginhoux F. Understanding the heterogeneity of resident liver macrophages. Front Immunol. (2019) 10:2694. 10.3389/fimmu.2019.02694 31803196 PMC6877662

[17] Krenkel O Tacke F. Liver macrophages in tissue homeostasis and disease. Nat Rev Immunol. (2017) 17:306–21. 10.1038/nri.2017.11 28317925

[18] MacParland SA Liu JC Ma XZ Innes BT Bartczak AM Gage BK. Single cell RNA sequencing of human liver reveals distinct intrahepatic macrophage populations. Nat Commun. (2018) 9:4383. 10.1038/s41467-018-06318-7 30348985 PMC6197289

[19] Aizarani N Saviano A Sagar M Durand S Herman JS Pessaux P. A human liver cell atlas reveals heterogeneity and epithelial progenitors. Nature. (2019) 572:199–204. 10.1038/s41586-019-1373-2 31292543 PMC6687507

[20] Wu X Roberto JB Knupp A Kenerson HL Truong CD Yuen SY. Precision-cut human liver slice cultures as an immunological platform. J Immunol Methods. (2018) 455:71–9. 10.1016/j.jim.2018.01.012 29408707 PMC6689534

[21] Mohar I Brempelis KJ Murray SA Ebrahimkhani MR Crispe IN. Isolation of non-parenchymal cells from the mouse liver. Methods Mol Biol. (2015) 1325:3–17. 10.1007/978-1-4939-2815-6_1 26450375

[22] Stuart SG Simister NE Clarkson SB Kacinski BM Shapiro M Mellman I. Human IgG Fc receptor. (hFcRII; CD32) exists as multiple isoforms in macrophages, lymphocytes and IgG-transporting placental epithelium. EMBO J. (1989) 8:3657–66. 10.1002/j.1460-2075.1989.tb08540.x 2531080 PMC402048

[23] Anania JC Chenoweth AM Wines BD Hogarth PM. The human FcgammaRII. (CD32) family of leukocyte FcR in health and disease. Front Immunol. (2019) 10:464. 10.3389/fimmu.2019.00464 30941127 PMC6433993

[24] Eischen A Vincent F Louis B Schmitt-Goguel M Bohbot A Bergerat JP Immunophenotypic characterisation of human peritoneal and alveolar macrophages and of human blood monocytes differentiated in the presence of either GM-CSF or M-CSF or a combination of GM-CSF/M-CSF. Nouvelle Revue Francaise D'hematologie. (1992) 34:421–34. 1300541

[25] van Schie RC Verstraten HG Tax WJ van den Berkmortel FW van de Winkel JG de Mulder PH. Effect of rIFN-gamma on antibody-mediated cytotoxicity via human monocyte IgG Fc receptor II. (CD32). Scand J Immunol. (1992) 36:385–93. 10.1111/j.1365-3083.1992.tb02952.x 1387726

[26] Onrust SV Lamb HM. Mometasone furoate. A review of its intranasal use in allergic rhinitis. Drugs. (1998) 56:725–45. 10.2165/00003495-199856040-00018 9806113

[27] Kocher M Siegel ME Edberg JC Kimberly RP. Cross-linking of Fc gamma receptor IIa and Fc gamma receptor IIIb induces different proadhesive phenotypes on human neutrophils. J Immunol. (1997) 159:3940–8. 9378982

[28] Fanger NA Wardwell K Shen L Tedder TF Guyre PM. Type I. (CD64) and type II. (CD32) Fc gamma receptor-mediated phagocytosis by human blood dendritic cells. J Immunol. (1996) 157:541–8. 8752900

[29] Sierro F Evrard M Rizzetto S Melino M Mitchell AJ Florido M. A liver capsular network of monocyte-derived macrophages restricts hepatic dissemination of intraperitoneal bacteria by neutrophil recruitment. Immunity. (2017) 47:374–88.e6. 10.1016/j.immuni.2017.07.018 28813662

[30] Ramachandran P Dobie R Wilson-Kanamori JR Dora EF Henderson BEP Luu NT. Resolving the fibrotic niche of human liver cirrhosis at single-cell level. Nature. (2019) 575:512–8. 10.1038/s41586-019-1631-3 31597160 PMC6876711

[31] Krenkel HO Ritz J Weiskirchen TP Tacke RF. Single cell RNA sequencing identifies subsets of hepatic stellate cells and myofibroblasts in liver fibrosis. Cells. (2019) 8:503. 10.3390/cells8050503 31137713 PMC6562512

[32] Bonnardel J T'Jonck J Gaublomme W Elewaut D Saeys D Guilliams YM. Stellate cells, hepatocytes and endothelial cells imprint the kupffer cell identify of monocytes colonizing the liver macrophage Niche. Immunity. (2019) 51:1–17. 10.1016/j.immuni.2019.08.017 31561945 PMC6876284

