# Supplementary material for: Human Liver Macrophage Subsets Defined by CD32
Source: Front Immunol. 2020 Sep 23;11:2108. doi: 10.3389/fimmu.2020.02108 (PMC7546764; doi:10.3389/fimmu.2020.02108)
Supplement: Supplementary file 1 [file Table_1.pdf]

Table S1 Patient information

Patient ID	Gender	Age	Diagnosis	Cell sorting and gene expression analysis	Flow analysis, BDCA1, BDCA3 panel	Flow analysis, CD16, CD206 panel
PT-1	F	67	Metastatic ovarian cancer	Y		
PT-2	F	38	Adenoma	Y		
PT-3	M	36	Cholangiocarcinoma	Y		
PT-4	M	71	Metastatic gastric cancer		Y	Y
PT-5	F	27	Metastatic colorectal cancer (mCRC)		Y	Y
PT-6	F	69	Metastatic high-grade serous carcinoma		Y	Y
PT-7	F	26	Adenoma		Y	Y
PT-8	M	54	mCRC		Y	Y
PT-9	F	60	Intrahepatic cholangiocarcinoma		Y	
PT-10	F	63	Metastatic duodenal gastrointestinal stromal tumors		Y	
PT-11	M	59	Cholangiocarcinoma		Y	
PT-12	F	26	Adenoma		Y	
PT-13	F	50	Metastatic gastrointestinal stromal tumor			Y
PT-14	M	33	Epithelioid hemangioendothelioma			Y
PT-15	F	32	Epithelioid hemangioendothelioma			Y
PT-16	F	55	mCRC			Y
PT-17	F	57	Metastatic granulosa cell tumor			Y
PT-18	M	72	mCRC			Y
PT-19	F	52	mCRC, mild steatosis			Y
PT-20	F	84	Hepatocellular carcinoma (HCC) and steatosis			Y
PT-21	F	50	mCRC			Y
PT-22	F	55	mCRC			Y
PT-23	F	47	mCRC			Y
PT-24	M	54	Metatatic pancreatic cancer			Y
PT-25	F	58	mCRC			Y
PT-26	M	57	mCRC			Y
