# Supplementary material for: Human Liver Macrophage Subsets Defined by CD32
Source: Front Immunol. 2020 Sep 23;11:2108. doi: 10.3389/fimmu.2020.02108 (PMC7546764; doi:10.3389/fimmu.2020.02108)
Supplement: Supplementary file 2 [file Table_2.pdf]

Table S2: list of all genes assayed by qRT-PCR

Assay #	Gene name	GeneBank Accession ID	NCBI protein ID	UniprotID
1	ACTB	NM_001101.3	NP_001092.1	P60709
2	GAPDH	NM_001256799.2	NP_001243728.1	P04406
3	HPRT1	NM_000194.2	NP_000185.1	P00492
4	TNF	NM_000594.3	NP_000585.2	P01375
5	IL-6	NM_000600.4	NP_000591.1	P05231
6	COL1A1	NM_000088.3	NP_000079.2	P02452
7	COL3A1	NM_000090.3	NP_000081.1	P02461
8	CTGF	NM_001901.2	NP_001892.1	P29279
9	MMP9	NM_004994.2	NP_004985.2	P14780
10	PDGFB	NM_002608.3	NP_002599.1	P01127
11	PDGFRA	NM_006206.4	NP_006197.1	P16234
12	TGFB1	NM_000660.5	NP_000651.3	P01137
13	TIMP1	NM_003254.2	NP_003245.1	P01033
14	CCL2	NM_002982.3	NP_002973.1	P13500
15	CCL3	NM_002983.2	NP_002974.1	P10147
16	CCL5	NM_002985.2	NP_002976.2	P13501
17	CCL7	NM_006273.3	NP_006264.2	P80098
18	CX3CL1	NM_002996.4	NP_002987.1	P78423
19	CXCL10	NM_001565.3	NP_001556.2	P02778
20	IL-8	NM_000584.3	NP_000575.1	P10145
21	CASP1	NM_001223.4	NP_001214.1	P29466
22	IL-1B	NM_000576.2	NP_000567.1	P01584
23	IL-18	NM_001243211.1	NP_001230140.1	Q14116
24	NLRP3	NM_001079821.2	NP_001073289.1	Q96P20
25	IFNA1	NM_024013.2	NP_076918.1	P01562
26	IFNB1	NM_002176.3	NP_002167.1	P01574
27	IFNG	NM_000619.2	NP_000610.2	P01579
28	IL-28B	NM_172139.2	NP_742151.2	Q8IZJ0
29	IFIT1	NM_001270927.1	NP_001257856.1	P09914
30	IFIT2	NM_001547.4	NP_001538.4	P09913
31	IFIT3	NM_001031683.3	NP_001026853.1	O14879
32	IL-12B	NM_002187.2	NP_002178.2	P29460
33	ISG15	NM_005101.3	NP_005092.1	P05161
34	ALB	NM_000477.5	NP_000468.1	P02768
35	FBP1	NM_000507.3	NP_000498.2	P09467

36	PDGFRB	NM_002609.3	NP_002600.1	P09619
37	SLC27A2	NM_001159629.1	NP_001153101.1	O14975
38	ACTA2	NM_001141945.2	NP_001135417.1	P62736
39	CLEC4F	NM_001258027.1	NP_001244956.1	Q8N1N0
40	CXCL13	NM_006419.2	NP_006410.1	O43927
41	EMR1	NM_001256252.1	NP_001243181.1	Q14246
42	LYVE1	NM_006691.3	NP_006682.2	Q9Y5Y7
43	NRP2	NM_003872.2	NP_003863.2	O60462
44	PTGS2	NM_000963.3	NP_000954.1	P35354
45	TIE1	NM_001253357.1	NP_001240286.1	P35590
46	CXCL1	NM_001511.3	NP_001502.1	P09341
47	CXCL2	NM_002089.3	NP_002080.1	P19875
48	CXCL9	NM_002416.2	NP_002407.1	Q07325
49	IL-1A	NM_000575.4	NP_000566.3	P01583
50	RSAD2	NM_080657.4	NP_542388.2	Q8WXG1
51	MX1	NM_001144925.2	NP_001138397.1	P20591
52	IL-12A	NM_000882.3	NP_000873.2	P29459
53	IL-10	NM_000572.2	NP_000563.1	P22301
54	SLC10A1	NM_003049.3	NP_003040.1	Q14973
55	CX3CR1	NM_001171171.1	NP_001164642.1	P49238
56	CD81	NM_001297649.1	NP_001284578.1	P60033
57	HLA-A	NM_001242758.1	NP_001229687.1	P30443
58	HLA-DRA	NM_019111.4	NP_061984.2	P01903
59	PD-L1	NM_001267706.1	NP_001254635.1	Q9NZQ7
60	OX40	NM_003327.3	NP_003318.1	P47741
61	4-1BB	NM_001561.5	NP_001552.2	Q07011
62	IDO1	NM_002164.5	NP_002155.1	P14902
63	CTLA4	NM_001037631.2	NP_001032720.1	P16410
64	CD47	NM_001777.3	NP_001768.1	Q08722
65	TIGIT	NM_173799.3	NP_776160.2	Q495A1
66	VISTA	NM_022153.1	NP_071436.1	Q9H7M9
67	CD40	NM_001250.5	NP_001241.1	P25942
68	CD80	NM_005191.3	NP_005182.1	P33681
69	CD86	NM_001206924.1	NP_001193853.1	P42081
70	CIITA	NM_000246.3	NP_000237.2	P33076
71	TNFSF9	NM_003811.3	NP_003802.1	P41273
72	TNFSF10	NM_001190942.1	NP_001177871.1	P50591
73	TIM3	NM_032782.4	NP_116171.3	Q8TDQ0
74	LAG3	NM_002286.5	NP_002277.4	P18627
75	ARG1	NM_000045.3	NP_000036.2	P05089
76	ICAM1	NM_000201.2	NP_000192.2	P05362
77	MARCO	NM_006770.3	NP_006761.1	Q9UEW3
78	PD-L2	NM_025239.3	NP_079515.2	Q9BQ51

79	TNFRSF18	NM_004195.2	NP_004186.1	Q9Y5U5
80	VCAM1	NM_001078.3	NP_001069.1	P19320
81	PDCD1	NM_005018.2	NP_005009.2	Q15116
82	FOXP3	NM_001114377.1	NP_001107849.1	Q9BZS1
83	FASLG	NM_000639.2	NP_000630.1	P48023
84	B7-H3	NM_001024736.1	NP_001019907.1	Q5ZPR3
85	B7-H4	NM_001253849.1	NP_001240778.1	Q7Z7D3
86	CCR2	NM_001123396.1	NP_001116868.1	P41597
87	TLR3	NM_003265.2	NP_003256.1	O15455
88	TLR4	NM_003266.3	NP_003257.1	O00206
89	TLR7	NM_016562.3	NP_057646.1	Q9NYK1
90	TLR8	NM_016610.3	NP_057694.2	Q9NR97
91	FCN1	AK314867.1	BAG37382.1	O00602
92	S100A12	NM_005621.1	NP_005612.1	P80511
93	MNDA	NM_002432.1	NP_002423.1	P41218
94	ITGA4	NM_000885.5	NP_000876.3	P13612
95	EMCN	NM_001159694.1	NP_001153166.1	Q9ULC0
96	VWF	NM_000552.4	NP_000543.2	P04275
97	ADGRL4	NM_022159.3	NP_071442.2	Q9HBW9
98	TEK	NM_000459.4	NP_000450.2	Q02763
99	CALCRL	NM_001271751.1	NP_001258680.1	Q16602
100	CD34	NM_001025109.1	NP_001020280.1	P28906
101	APOB	NM_000384.2	NP_000375.2	P04114
102	UGT2B7	NM_001074.2	NP_001065.2	P16662
103	PAH	NM_000277.1	NP_000268.1	P00439
104	CYP3A5	NM_000777.4	NP_000768.1	P20815
105	IGFBP1	NM_000596.2	NP_000587.1	P08833
106	FGFR2	NM_000141.4	NP_000132.3	P21802
107	AFM	NM_001133.2	NP_001124.1	P43652
108	MRC1	NM_002438.3	NP_002429.1	P22897
109	VCAN	NM_001126336.2	NP_001119808.1	P13611
110	CD163	NM_004244.5	NP_004235.4	Q86VB7
111	CD68	NM_001040059.1	NP_001035148.1	P34810
112	CD5L	NM_005894.2	NP_005885.1	O43866
113	FCGR2B	NM_001002273.2	NP_001002273.1	P31994
114	FCGR2A	NM_021642.3	NP_067674.2	P12318
115	VSIG4	NM_001100431.1	NP_001093901.1	Q9Y279
116	LYZ	NM_000239.2	NP_000230.1	P61626
117	S100A8	NM_001319196.1	NP_001306125.1	P05109
118	S100A9	NM_002965.3	NP_002956.1	P06702
119	HMOX1	NM_002133.2	NP_002124.1	P09601
120	MERTK	NM_006343.2	NP_006334.2	Q12866
121	FIZZ1	NM_032579.2	NP_115968.1	Q9BQ08

122	IL4	NM_000589.3	NP_000580.1	P05112
123	IL13	NM_002188.2	NP_002179.2	P35225
124	IL4R	NM_000418.3	NP_000409.1	P24394
125	LRAT	NM_001301645.1	NP_001288574.1	O95237
126	CYP1B1	NM_000104.3	NP_000095.2	Q16678
127	CYP26A1	NM_000783.3	NP_000774.2	O43174
128	AOX1	NM_001159.3	NP_001150.3	Q06278
129	RBP4	NM_001323517.1	NP_001310446.1	P02753
130	RBP1	NM_001130992.1	NP_001124464.1	P09455
131	STRA6	NM_001142617.1	NP_001136089.1	Q9BX79
132	CYP3A4	NM_001202855.2	NP_001189784.1	P08684
133	CYP2C8	NM_000770.3	NP_000761.3	P10632
134	ALDH1A1	NM_000689.4	NP_000680.2	P00352
135	ADH7	NM_000673.4	NP_000664.2	P40394
136	CRABP1	NM_004378.2	NP_004369.1	P29762
137	RPE65	NM_000329.2	NP_000320.1	Q16518
138	ALDH1A2	NM_001206897.1	NP_001193826.1	O94788
139	SPHK1	NM_001142601.1	NP_001136073.1	Q9NYA1
140	SOX9	NM_000346.3	NP_000337.1	P48436
