# Supplementary figures and images for: Human Liver Macrophage Subsets Defined by CD32
Source: Front Immunol. 2020 Sep 23;11:2108. doi: 10.3389/fimmu.2020.02108 (PMC7546764; doi:10.3389/fimmu.2020.02108)

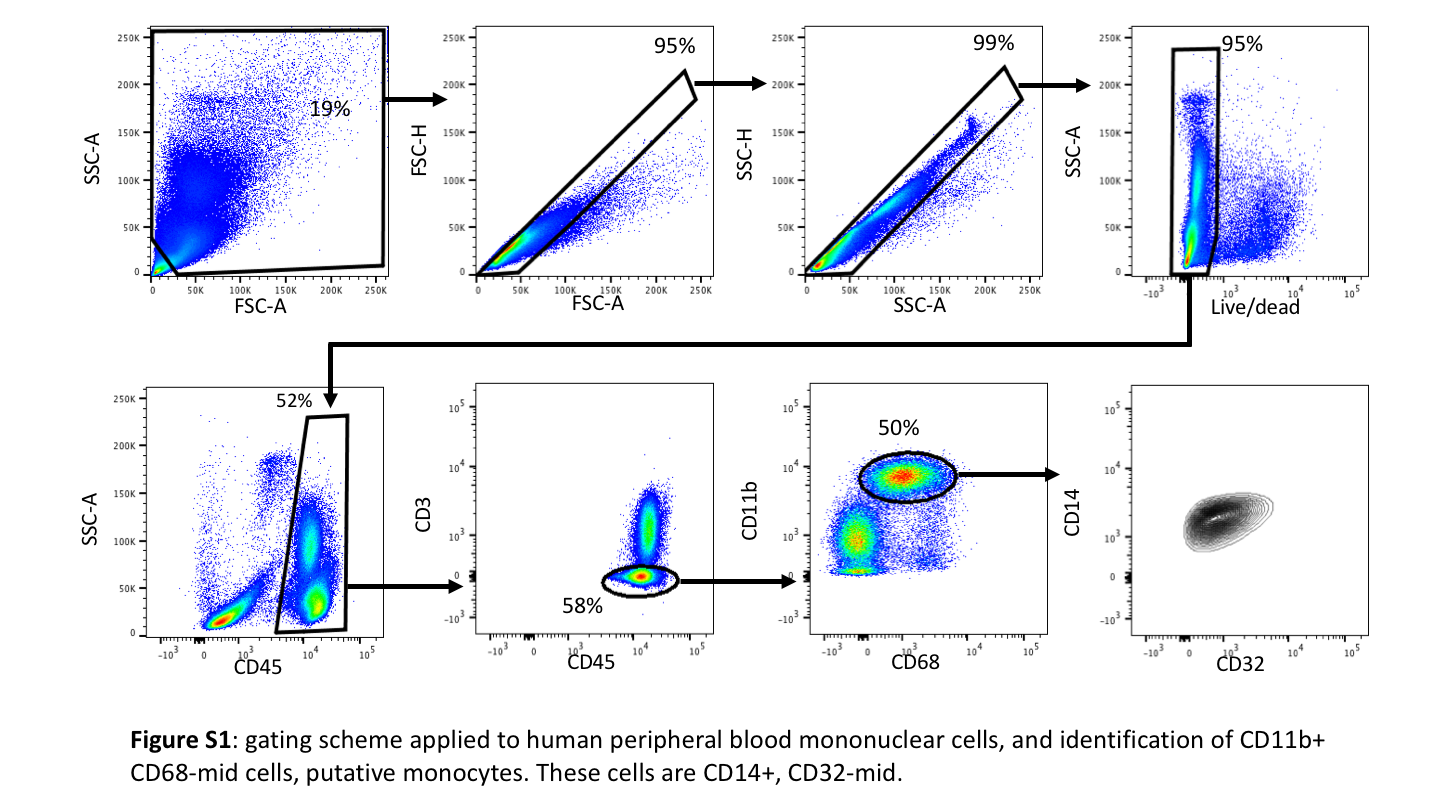

Supplement: Supplementary file 3 [file Data_Sheet_1.zip › FigureS1_revised.tiff]

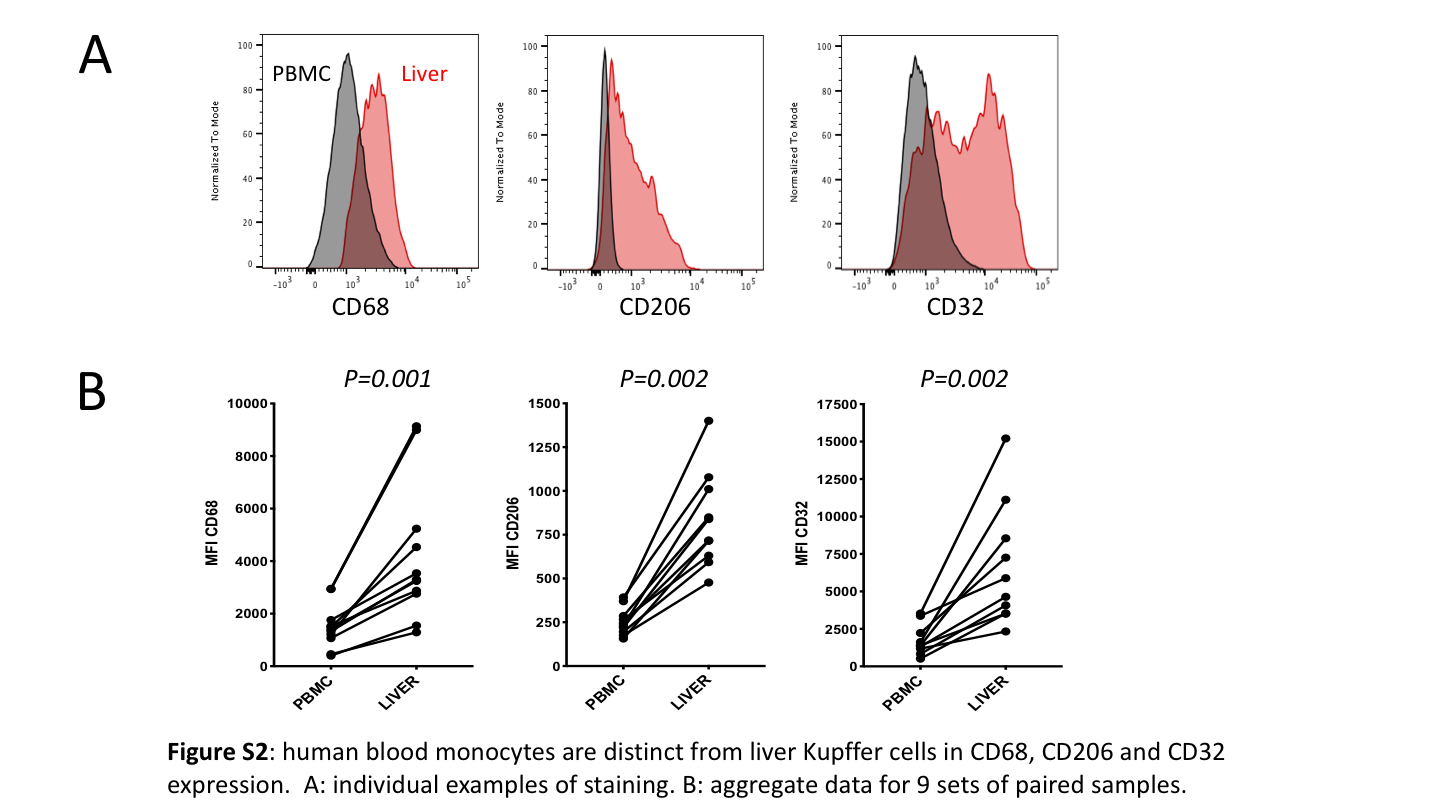

Supplement: Supplementary file 3 [file Data_Sheet_1.zip › FigureS2_revised.tiff]

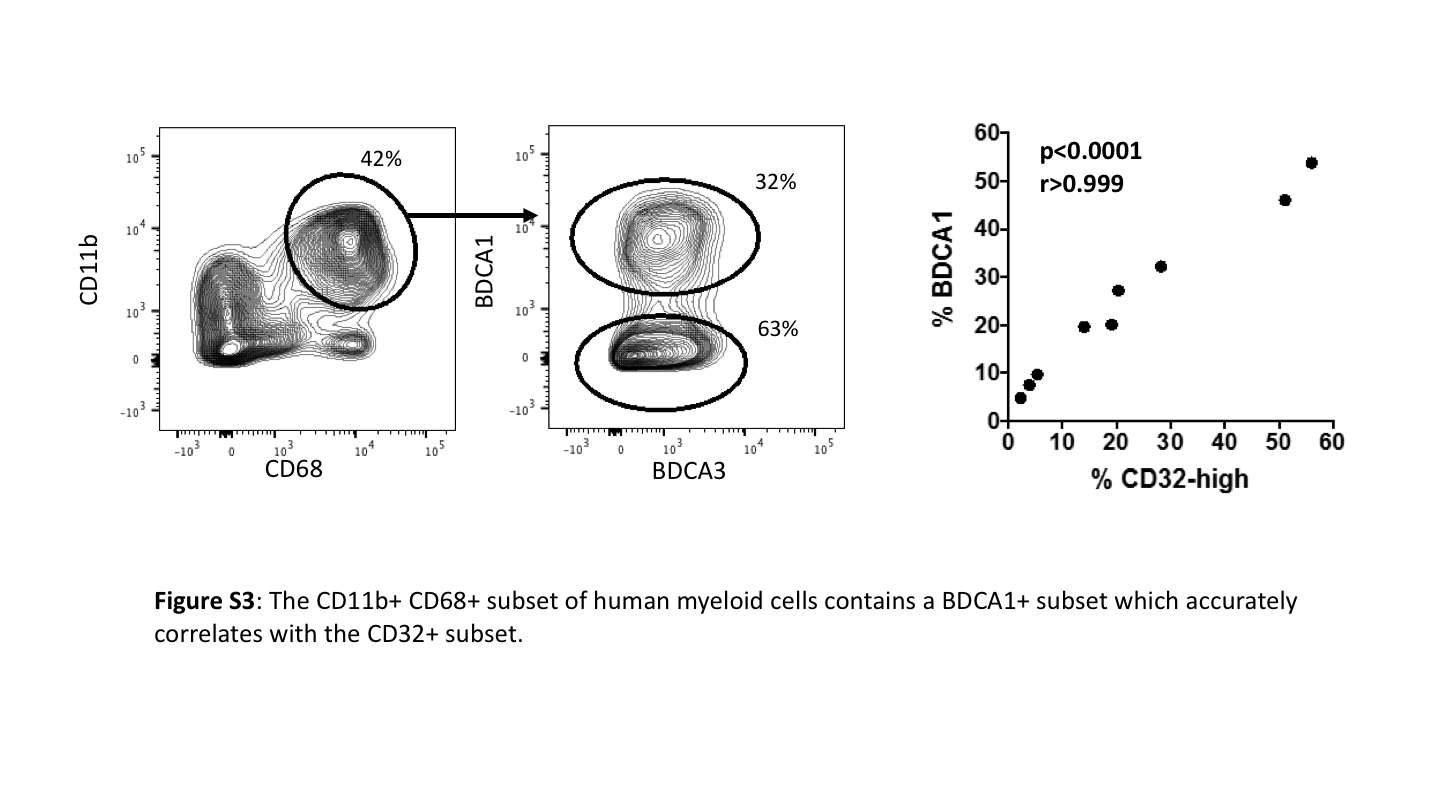

Supplement: Supplementary file 3 [file Data_Sheet_1.zip › FigureS3_revised.tiff]

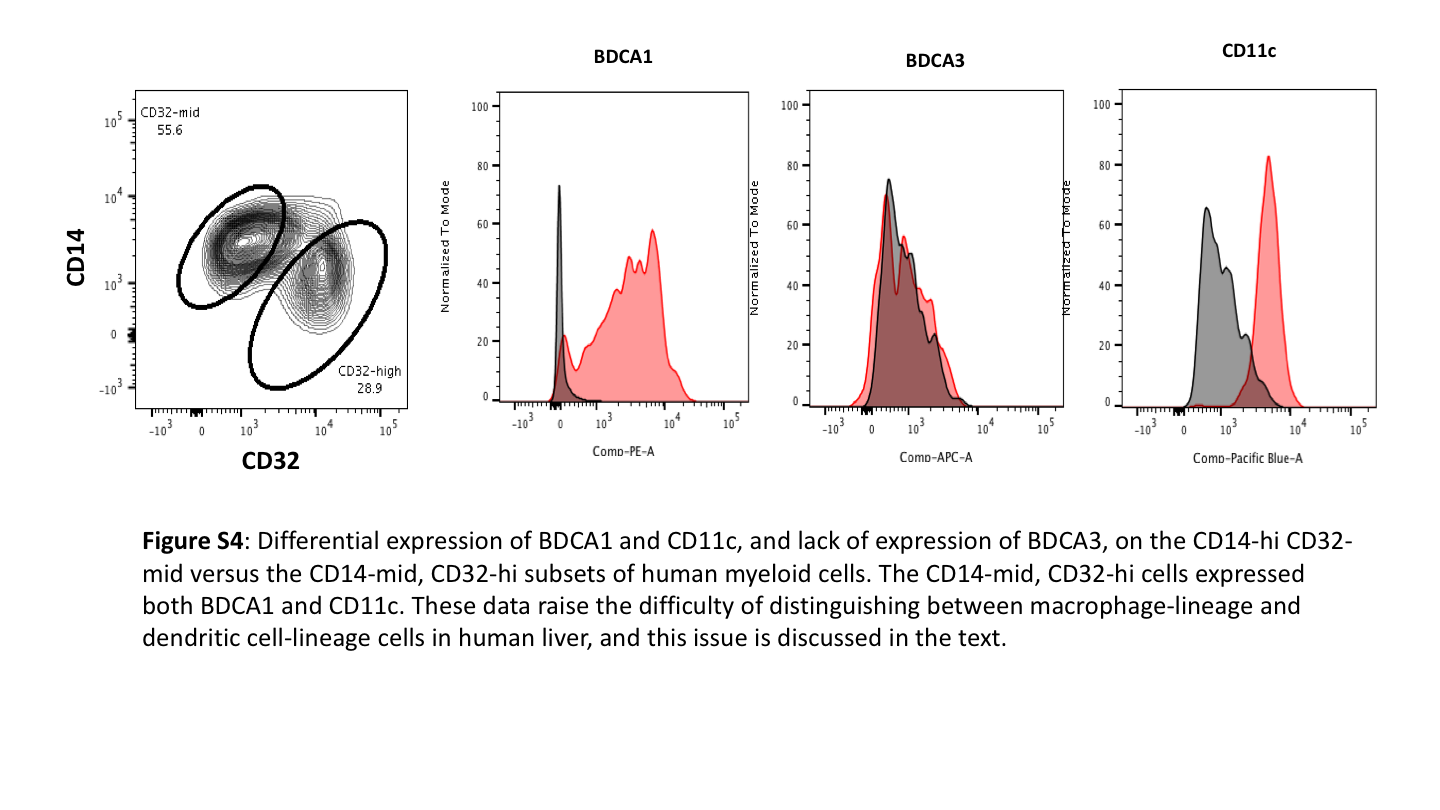

Supplement: Supplementary file 3 [file Data_Sheet_1.zip › FigureS4_revised.tiff]

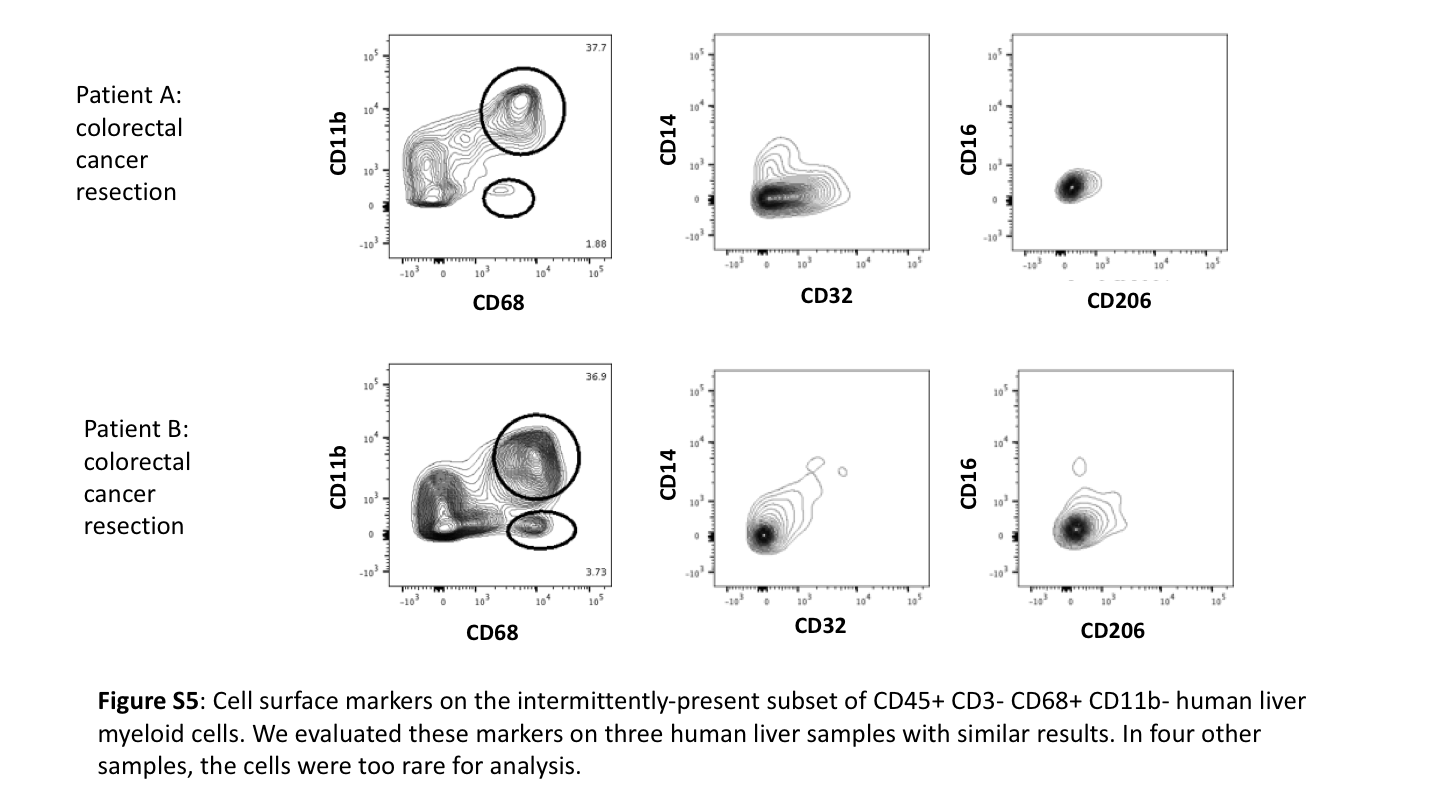

Supplement: Supplementary file 3 [file Data_Sheet_1.zip › FigureS5_revised.tiff]
